# Switching to Lurasidone following 12 months of treatment with Risperidone: results of a 6-month, open-label study

**DOI:** 10.1186/s12888-020-02523-1

**Published:** 2020-05-05

**Authors:** Greg W. Mattingly, Peter M. Haddad, Michael Tocco, Jane Xu, Debra Phillips, Andrei Pikalov, Antony Loebel

**Affiliations:** 1 Washington University School of Medicine, St. Louis, MO USA; 2 Hamad Medical Corporation, Doha, Qatar; 3 Clinical Professor of Psychiatry, Qatar University, Doha, Qatar; 4 Honorary Professor of Psychiatry, University of Manchester, Manchester, UK; 5 Honorary Consultant Psychiatrist, GMMH NHS Foundation Trust, Manchester, UK; 6 Sunovion Pharmaceuticals Inc., Fort Lee, NJ, 84 Waterford Dr, Marlborough, MA 01752 USA

**Keywords:** Lurasidone, Antipsychotic agents, Schizophrenia, Adverse effects, Weight, Metabolic, Lipids, Prolactin

## Abstract

**Background:**

Patients with a diagnosis of schizophrenia are at an increased risk for developing metabolic syndrome, which is associated with greater cardiovascular morbidity and mortality. Treatment with some commonly used antipsychotic medications may increase the risk of developing metabolic syndrome. The aim of the study was to evaluate the safety of lurasidone in patients who continued lurasidone or switched from risperidone to lurasidone. A secondary aim was assessment of the effect of long-term lurasidone on the Positive and Negative Syndrome Scale (PANSS).

**Methods:**

The treatment sample in the current study consisted of clinically stable patients with schizophrenia (*N* = 223) who had completed a 12-month, double-blind study of lurasidone vs. risperidone. In the current extension study, all patients received 6 months of open-label treatment with lurasidone, either continuing lurasidone assigned during the preceding double-blind trial, or switching from double-blind risperidone to lurasidone. Safety and tolerability parameters included body weight, prolactin, and metabolic laboratory tests.

**Results:**

Six months of OL treatment with lurasidone was generally well-tolerated, with a low incidence of parkinsonism (4.5%) and akathisia (3.1%). Overall, few adverse events were rated as severe (4.9%), and discontinuation due to an adverse event was low in the lurasidone continuation vs. risperidone switch groups (3.7% vs. 6.9%). In the lurasidone continuation versus risperidone switch groups, change from OL baseline to 6-month endpoint (observed case) was observed in mean body weight (− 0.6 vs. -2.6 kg), median total cholesterol (− 4.0 vs. + 4.5 mg/dL), triglycerides (− 4.5 vs. -5.5 mg/dL), glucose (0.0 vs. -3.0 mg/dL) and prolactin (males, + 0.15 vs. -11.2 ng/mL; females, + 1.3 vs. -30.8 ng/mL). Improvement in PANSS total score was maintained, from OL baseline to endpoint in the continuation vs. switch groups (+ 1.0 vs. -1.0; OC).

**Conclusions:**

In this 6-month extension study, lurasidone treatment was generally well-tolerated and associated with minimal effects on weight, metabolic parameters, and prolactin levels. Patients who switched from risperidone to lurasidone experienced reductions in weight, metabolic parameters and prolactin levels commensurate with increases in these safety parameters experienced during the previous 12 months of treatment with risperidone.

**Trial registration:**

ClinicalTrials.gov NCT00641745 (Date of Registration: March 24, 2008).

## Background

Non-response to treatment with an initial antipsychotic occurs in at least 50% of patients with first episode schizophrenia and increases as the illness becomes more chronic and recurrent [1, 2]. The recommended next-step treatment option in non-responders is switching to an alternative antipsychotic [3, 4]. In addition to lack of efficacy, problems with safety or tolerability frequently necessitate switching antipsychotics [5].

Lurasidone is an atypical antipsychotic agent that has demonstrated efficacy in short-term [6–9] and long-term studies [10–12] of patients with schizophrenia, with a safety profile indicating minimal effects on weight, metabolic parameters, and prolactin [13, 14].

Previously, the effectiveness of switching patients with schizophrenia or schizoaffective disorder to lurasidone using 3 different dosing strategies has been evaluated [15]. At the time of the switch, patients were in a non-acute phase of their illness and were being treated with a wide range of typical or atypical antipsychotics. This 6-week study demonstrated that switching patients to lurasidone was associated with good efficacy and tolerability and low rates of treatment failure (8%), regardless of switching strategy (rapid or slow titration of lurasidone). Initial improvement in weight and lipids was observed after 6 weeks of treatment. In a 6-month, open-label extension of this study, improvements in efficacy on lurasidone were maintained, with minimal long-term effects on weight, metabolic parameters, and prolactin [16].

The effect on safety parameters of switching patients with schizophrenia from olanzapine to lurasidone has also been evaluated in a 6-month, open-label extension study in which patients who completed 6 weeks of double-blind, placebo-controlled treatment with olanzapine or lurasidone were switched to 6 months of open-label lurasidone 40–120 mg/d [17]. At 6-month endpoint, switching from olanzapine to lurasidone resulted in clinically meaningful (≥7%) reduction in weight in 29.0% of patients; and median reduction in lipid parameters, including total cholesterol (− 15.0 mg/dL) and triglycerides (− 28.0 mg/dL).

We now report results of an open-label extension study in which patients with schizophrenia who completed a double-blind, 12-month study of lurasidone versus risperidone [18] either continued lurasidone or switched from risperidone to lurasidone for an additional 6 months of open-label treatment. Notable safety results for lurasidone vs. risperidone at endpoint of the initial double-blind study included: mean reduction in weight (− 1.0 vs. + 1.5 kg) and waist circumference (− 0.6 vs. + 1.6 cm); smaller mean increases in prolactin for females (+ 34.9 vs. 53.3 ng/mL) but similar increases for males (13.5 vs. 14.1 ng/mL).

The primary objective of this study was to evaluate the long-term safety, tolerability and overall effectiveness of lurasidone in both the continuation and risperidone switch groups.

## Methods

### Study design

Detailed methods for the initial 12-month, double-blind study have been previously reported [18]. Briefly, clinically stable outpatients, ages 18–75 years, with a diagnosis of schizophrenia or schizoaffective disorder, were randomly assigned in a 2:1 ratio to receive lurasidone (flexibly dosed, 40–120 mg/d) or risperidone (flexibly dosed, 2–6 mg/d). Study completers were eligible to continue into the current 6-month, open-label extension study that was conducted from March 2009 to January 2011 at sites in the United States (*n* = 40), South Africa (*n* = 7), Argentina (*n* = 5), Chile (*n* = 5), Brazil (*n* = 4), Croatia (*n* = 3), Thailand (*n* = 3), and Israel (*n* = 1). To maintain the double-blind in the initial 12-month study, all patients entering the current open-label study received 3 days of single-blind placebo washout followed by 7 days of lurasidone 80 mg/d. After 7 days, the lurasidone dose could be titrated, based on the judgment of the investigator, in the range of 40–120 mg/d.

The study was conducted in accordance with the Good Clinical Practice Guidelines of the International Conference on Harmonisation and with the ethical principles of the Declaration of Helsinki. The study was approved by an institutional review board or independent ethics committee at each study site, and all patients provided written informed consent prior to initiation of study procedures. No important changes in study design or methodology were made after the study was initiated.

### Assessments

Assessment visits occurred at baseline of the open-label extension study and monthly thereafter. Adverse events were based on patient self-report in response to an open-ended question or were based on investigator observation of changes in the patient during examination. Movement disorder symptoms were evaluated with the Simpson-Angus Scale (SAS) [19], Barnes Akathisia Rating Scale (BARS) [20], and Abnormal Involuntary Movement Scale (AIMS) [21]. Safety assessments included laboratory tests (chemistry and hematology panels, lipid panel, glycosylated hemoglobin [HbA1c], bone alkaline phosphatase, N-telopeptide, osteocalcin, parathyroid hormone, prolactin, and testosterone), electrocardiograms (ECG), physical examinations, and vital sign measurements. In a subset of patients (at selected US sites), bone mineral density assessments were performed (BMD, using dual-energy x-ray absorptiometry [DXA]). T-scores were calculated ([patient’s BMD – mean BMD of sex-matched young adults] / 1-SD of young adults), and standard criteria were used to determine BMD category (normal vs. osteopenia vs. osteoporosis) [22]. Ophthalmologic examinations, including dilated funduscopic and slit lamp eye examinations, were also performed.

Efficacy was assessed using the Positive and Negative Syndrome Scale (PANSS) [23], Clinical Global Impression, Severity scale [21], and the Montgomery-Åsberg Depression Rating Scale (MADRS) [24]. Training and certification of raters at each investigational site on study assessments was provided prior to initiation of the double-blind study.

### Statistical analysis

The primary safety analysis population consisted of all patients who received at least one dose of lurasidone during the 6-month open-label extension study. All safety and efficacy outcomes were pre-specified and were analyzed for the overall treatment sample, and for 2 patient subgroups: patients who received lurasidone in the double-blind study, and patients who received risperidone in the double-blind study. Change scores were calculated from double-blind baseline to open-label study endpoint and from open-label baseline to open-label study endpoint (month 6). Observed cases (OC) and last observation carried forward (LOCF-endpoint) analyses were performed.

## Results

### Patient disposition and study treatment

**Fig. 1:**
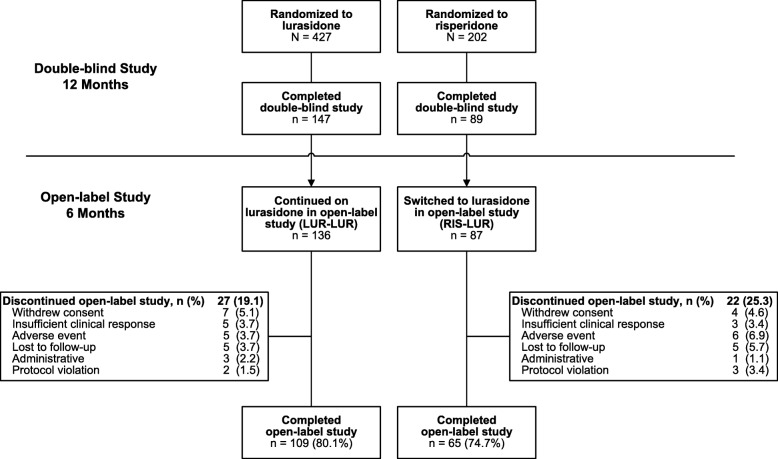
Patient Disposition. LUR = lurasidone; RIS = risperidone; DB: double-blind; OLE: open-label extension

Of the 236 patients who completed the initial 12-month double-blind study, 223 (94.5%) continued into the current open-label extension study. Overall, 90.1% of patients completed at least 3 months of open-label treatment with lurasidone, and 174/223 (78.0%) completed 6 months of treatment. Reasons for premature discontinuation included adverse events (11/223; 4.9%), withdrew consent (11/223; 4.9%), lost to follow-up (10/223; 4.5%), insufficient clinical response (8/223; 3.6%), and miscellaneous other reasons (9/223; 4.0%). Figure 1 summarizes patient disposition for the two pre-specified patient subgroups (based on double-blind treatment assignment in the initial double-blind study.

Patient characteristics were similar at open-label baseline in both the lurasidone continuation subgroup, and the risperidone-to-lurasidone switch subgroup (Table 1). The mean daily dose of lurasidone during open-label extension was 81.1 mg. Twenty-nine percent of patients (*n* = 65) received at least one concomitant medication, most commonly anxiolytics (22%), hypnotics/sedatives (18%), antidepressants (15%), and anticholinergics (13%).

**Table 1:** Patient Characteristics (Open-Label Baseline, Safety Population)

Characteristic	LUR-LUR^a^ (*N* = 136)	RIS-LUR^b^ (*N* = 87)
Male, n (%)	102 (75.0)	58 (66.7)
Age, mean (SD), y	43.9 (10.7)	42.8 (10.8)
Race, n (%)		
White	50 (36.8)	39 (44.8)
Black	67 (49.3)	40 (46.0)
Asian	6 (4.4)	1 (1.1)
Other	13 (9.6)	7 (8.0)
Ethnicity, Hispanic/Latino, n (%)	36 (26.5)	25 (28.7)
Duration of illness, mean (SD), y	16.9 (10.7)	17.6 (11.9)
≥4 hospitalizations, n (%)	30 (22.1)	25 (28.8)
PANSS total score, mean (SD)	55.4 (13.6)	55.5 (11.2)
CGI-S score, mean (SD)	2.8 (0.8)	2.9 (0.8)
MADRS score, mean (SD)	5.1 (5.6)	4.3 (4.4)

*CGI-S* Clinical Global Impression-Severity Scale, *LUR* lurasidone, *MADRS* Montgomery-Åsberg Depression Rating Scale, *PANSS* Positive and Negative Syndrome Scale, *RIS* risperidone, *SD* standard deviation

^a^ Patients who received lurasidone in both double-blind and open-label studies

^b^ Patients who received risperidone during the double-blind study and were switched to lurasidone in the open-label study

### Safety

#### Adverse events

The most commonly reported adverse events were headache (6.3%), psychotic disorder (5.4%), and parkinsonism (4.5%; Table 2), with minimal differences between the lurasidone continuation versus risperidone switch groups. For both groups combined, a total of 11 patients (4.9%) experienced an adverse event rated as severe; and 10 patients (4.5%) experienced a serious adverse event, consisting of schizophrenia (*n* = 3), psychotic disorder (*n* = 3), ankle fracture (*n* = 1), lung carcinoma (*n* = 1), possible seizure (*n* = 1), attempted suicide (*n* = 1; patient recovered and completed the study), and a completed suicide (*n* = 1; on open-label day 22 in a patient who had previously received 12 months of double-blind lurasidone, and who was experiencing recurrent psychotic symptoms).

**Table 2:** Adverse Events Reported in ≥2% of Patients During Open-Label Treatment With Lurasidone

Adverse Event, n (%)	LUR-LUR^a^ (*N* = 136)	RIS-LUR^b^ (*N* = 87)
≥1 adverse event	80 (58.8)	51 (58.6)
Headache	7 (5.1)	7 (8.0)
Psychotic disorder	6 (4.4)	6 (6.9)
Parkinsonism	5 (3.7)	5 (5.7)
Anxiety	2 (1.5)	6 (6.9)
Blood creatine phosphokinase increased	5 (3.7)	3 (3.4)
Insomnia	3 (2.2)	5 (5.7)
Nasopharyngitis	5 (3.7)	3 (3.4)
Akathisia	5 (3.7)	2 (2.3)
Somnolence	5 (3.7)	2 (2.3)
Influenza	6 (4.4)	1 (1.1)
Nausea	3 (2.2)	3 (3.4)
Upper respiratory infection	6 (4.4)	0 (0)
Vomiting	3 (2.2)	3 (3.4)
Back pain	2 (1.5)	3 (3.4)
Decreased appetite	3 (2.2)	2 (2.3)
Weight decreased	4 (2.9)	1 (1.1)

*LUR* lurasidone, *RIS* risperidone

^a^ Patients who received lurasidone in both double-blind and open-label studies

^b^ Patients who received risperidone during the double-blind study and were switched to lurasidone in the open-label study

#### Extrapyramidal symptoms

In the combined patient groups, the proportion who reported an extrapyramidal symptom (EPS)-related adverse event during the extension study was 7.6%, and the proportion with akathisia was 3.1%. EPS-related adverse events reported in more than 1 patient were parkinsonism (4.5%) and dystonia (1.3%). The incidence of an EPS-related adverse event was similar in the lurasidone continuation versus risperidone switch groups (Table 2). No patient discontinued due to an EPS-related adverse event or akathisia. Mean change from open-label baseline to study endpoint (LOCF) was 0.0 on the Simpson-Angus Scale, 0.0 on the Barnes Akathisia Rating Scale global clinical assessment of akathisia, and + 0.3 on the Abnormal Involuntary Movement Scale total score.

#### Body weight, BMI, waist circumference

**Fig. 2:**
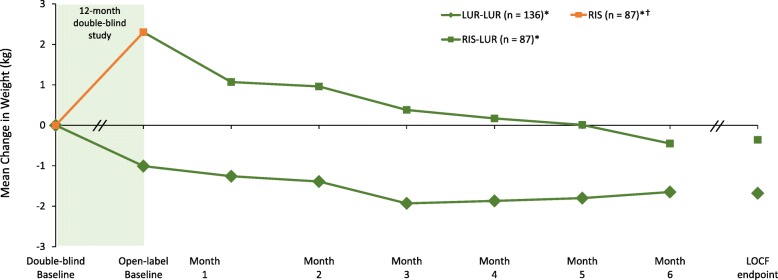
Median Change in Weight From Double-Blind Baseline Through 6 Months of Open-Label Treatment With Lurasidone, by Treatment Assignment in the Double-Blind Study. LUR = lurasidone; RIS = risperidone. ^*^Subgroup entering open-label extension; 6-month completer analysis: LUR-LUR, *n* = 109; RIS-LUR, *n* = 66. ^†^Patients in the RIS-LUR group received risperidone in the 12-month, double-blind study

Mean weight, BMI, and waist circumference were reduced, from double-blind to open-label baseline, in patients who received 12 months of treatment with lurasidone (− 1.1 kg, − 0.55 kg/m^2^, and − 0.4 cm, respectively), and were increased in patients who received 12 months of treatment with risperidone (+ 2.4 kg, + 2.1 kg/m^2^, + 2.8 cm, respectively; Table 3**;** Fig. 2).

**Table 3:** Change From Double-blind Baseline in Safety Parameters After 12-months of Treatment With Lurasidone or Risperidone, Followed by 6-months of Open-label Treatment With Lurasidone (OC analysis)

Parameter	LUR-LUR	RIS-LUR
**Weight, kg**	***n*** **= 109**^**a**^	***n*** **= 66**^**a**^
DB Baseline mean (SD)	81.1 (18.25)	82.9 (18.65)
Mean change to OL Baseline (after 12-mo DB Tx)	−1.1	+ 2.4
Mean change from OL Baseline to Month 6-OL	−0.6	−2.9
≥ 7% weight increase from DB Baseline, %	12.8	13.6
≥ 7% weight decrease from DB Baseline, %	28.4	18.2
≥ 7% weight increase from OL Baseline, %	1.8	3.0
≥ 7% weight decrease from OL Baseline, %	6.4	19.7
**Body mass index, kg/m**^**2**^	***n*** **= 109**	***n*** **= 66**
DB Baseline mean (SD)	27.7 (5.3)	28.8 (5.6)
Mean change to OL Baseline (after 12-mo DB Tx)	−0.55	+ 2.1
Mean change from OL Baseline to Month 6-OL	−0.2	−1.0
**Waist circumference, cm**	***n*** **= 104**	***n*** **= 62**
DB Baseline mean (SD)	93.8 (14.1)	97.5 (14.3)
Mean change to OL Baseline (after 12-mo DB Tx)	−0.4	+ 2.8
Mean change from OL Baseline to Month 6-OL	−0.9	−1.6
**Total cholesterol, mg/dL**	***n*** **= 108**	***n*** **= 64**
DB Baseline mean (SD)	196.4 (45.4)	188.0 (49.0)
Median change to OL Baseline (after 12-mo DB Tx)	−8.5	−9.0
Median change from OL Baseline to Month 6-OL	−4.0	+ 4.5
**Triglycerides, mg/dL**	***n*** **= 108**	***n*** **= 64**
DB Baseline mean (SD)	127.5 (57.7)	125.5 (88.8)
Median change to OL Baseline (after 12-mo DB Tx)	−13.0	+ 1.0
Median change from OL Baseline to Month 6-OL	−4.5	−5.5
**Glucose, mg/dL**	***n*** **= 105**	***n*** **= 63**
DB Baseline mean (SD)	95.1 (14.5)	94.6 (13.7)
Median change to OL Baseline (after 12-mo DB Tx)	−1.0	+ 3.0
Median change from OL Baseline to Month 6-OL	0.0	−3.0
**Hemoglobin A1c, %**	***n*** **= 103**	***n*** **= 63**
DB Baseline mean (SD)	5.7 (0.4)	5.6 (0.4)
Median change to OL Baseline (after 12-mo DB Tx)	0.0	0.0
Median change from OL Baseline to Month 6-OL	0.0	0.0
**Bone alkaline phosphatase, mcg/L**	***n*** **= 106**	***n*** **= 61**
DB Baseline mean (SD)	13.6 (5.2)	13.9 (4.3)
Median change to OL Baseline (after 12-mo DB Tx)	−0.9	− 0.3
Median change from OL Baseline to Month 6-OL	+ 1.5	0
**N-telopeptide (urine), nmol BCE/mmol creatinine**	***n*** **= 104**	***n*** **= 62**
DB Baseline mean (SD)	41.2 (120.3)	37.0 (35.8)
Median change to OL Baseline (after 12-mo DB Tx)	+ 1.5	−4.0
Median change from OL Baseline to Month 6-OL	−1.0	+ 0.5
**Osteocalcin, ng/mL**	***n*** **= 104**	***n*** **= 61**
DB Baseline mean (SD)	5.25 (3.38)	5.70 (4.36)
Median change to OL Baseline (after 12-mo DB Tx)	−0.85	−1.0
Median change from OL Baseline to Month 6-OL	0	0
**Parathyroid hormone, pg/mL**	***n*** **= 105**	***n*** **= 61**
DB Baseline mean (SD)	38.6 (17.4)	43.2 (27.8)
Median change to OL Baseline (after 12-mo DB Tx)	0	−2.0
Median change from OL Baseline to Month 6-OL	+ 2.0	+ 4.0
**Prolactin, ng/mL, males**	***n*** **= 84**	***n*** **= 43**
DB Baseline mean (SD)	7.7 (6.7)	10.2 (6.5)
Median change to OL Baseline (after 12-mo DB Tx)	−0.6	+ 12.8
Median change from OL Baseline to Month 6-OL	+ 0.15	−11.2
**Prolactin, ng/mL, females**	***n*** **= 24**	***n*** **= 21**
DB Baseline mean (SD)	20.0 (24.7)	18.6 (40.8)
Median change to OL Baseline (after 12-mo DB Tx)	−0.75	+ 35.2
Median change from OL Baseline to Month 6-OL	+ 1.3	−30.8
**Testosterone, total, ng/dL, males**	***n*** **= 84**	***n*** **= 42**
DB Baseline mean (SD)	498.1 (198.4)	481.3 (231.5)
Median change to OL Baseline (after 12-mo DB Tx)	+ 24.9	−103.0
Median change from OL Baseline to Month 6-OL	−23.5	+ 43.5
**Testosterone, free, pg/mL, males**	***n*** **= 84**	***n*** **= 40**
DB Baseline mean (SD)	10.3 (5.5)	9.6 (6.2)
Median change to OL Baseline (after 12-mo DB Tx)	−0.015	−1.405
Median change from OL Baseline to Month 6-OL	−1.06	−0.095

*BCE* bone collagen equivalent, *LUR* lurasidone, *RIS* risperidone, *DB* double-blind, *OL* open-label, *SD* standard deviation, *OC* observed case

^**a**^ Results presented are an observed case analysis of the number of patients available with test results at Month 18

Mean changes in mean weight, BMI, and waist circumference at 6-month open-label endpoint (OC analysis) were minimal in the lurasidone continuation subgroup; in contrast, notable reductions were observed in the subgroup that switched from risperidone to lurasidone (− 2.9 kg, − 1.0 kg/m^2^, − 1.6 cm, respectively; [OC]); and the proportion of patients who experienced ≥7% weight loss was 19.7%; Table 3).

#### Metabolic parameters

Median total cholesterol, triglycerides, and glucose were reduced, from double-blind to open-label baseline, in patients who received 12 months of treatment with lurasidone (− 8.5 mg/dL, − 13.0 mg/dL, − 1.0 mg/dL, respectively); and in patients who received 12 months of treatment with risperidone, median triglycerides and glucose were minimally increased (+ 1.0 mg/dL, + 3.0 mg/dL, respectively), while total cholesterol was reduced (− 9.0 mg/dL; Table 3). Median hemoglobin A1c levels were unchanged at double-blind endpoint in both treatment groups.

In the lurasidone continuation group, minimal changes were observed at 6-month open-label endpoint in median total cholesterol, triglycerides, glucose, and hemoglobin A1c (Table 3). In the risperidone switch group, small reductions were observed in triglycerides and glucose from open-label baseline to 6-month endpoint (− 5.5 mg/dL, − 3.0 mg/dL, respectively; OC); while total cholesterol increased (+ 4.5 mg/dL; Table 3). Median hemoglobin A1c levels were unchanged from open-label baseline to 6-month endpoint in both patient groups.

#### Prolactin

**Fig. 3:**
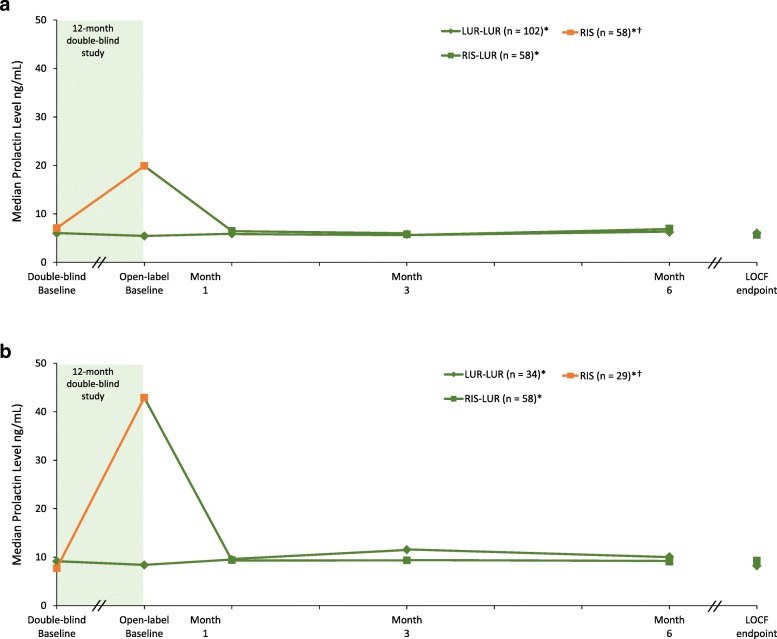
Median Change in Prolactin From Double-Blind Baseline Through 6 Months of Open-Label Treatment With Lurasidone, by Treatment Assignment in the Double-Blind Study. 3-A. Males. LUR = lurasidone; RIS = risperidone. ^*^Subgroup entering open-label extension; 6-month completer analysis: LUR-LUR, *n* = 102; RIS-LUR, *n* = 68. ^†^Patients in the RIS-LUR group received risperidone in the 12-month, double-blind study. 3-B. Females. LUR = lurasidone; RIS = risperidone. ^*^Subgroup entering open-label extension; 6-month completer analysis: LUR-LUR, *n* = 34; RIS-LUR, *n* = 29. ^†^Patients in the RIS-LUR group received risperidone in the 12-month, double-blind study

Median change in prolactin were notably different, from double-blind to open-label baseline, after 12 months of double-blind treatment with lurasidone and risperidone in both men (− 0.6 ng/mL vs. + 12.8 ng/mL), and women (− 0.75 ng/mL vs. + 35.2 ng/mL). In the lurasidone continuation group, median change in prolactin was minimal, from open-label baseline to 6-month endpoint (OC analysis), for men (+ 0.15 ng/mL) and women (+ 1.3 ng/mL); in the risperidone switch group notable reductions were observed after 6 months of treatment with lurasidone for men (− 11.2 ng/mL) and women (− 30.8 ng/mL; Table 3**;** Fig. 3a and b). No galactorrhea, amenorrhea or gynecomastia were observed in patients treated with open-label lurasidone.

#### Bone turnover markers and bone mineral density

As summarized in Table 3, minimal changes were observed in markers of bone turnover (bone alkaline phosphatase, osteocalcin, bone collagen equivalents, and urinary N-telopeptide) for both lurasidone and risperidone during 12 months of double-blind treatment, and during 6 months of open-label treatment with lurasidone.

In a subset of patients at US sites, bone mineral density (BMD) was assessed by dual-energy x-ray absorptiometry [DXA]. Based on DXA assessments, no loss of bone mineral density was observed during 6 months of open-label treatment with lurasidone in either the lurasidone continuation group (*n* = 46) or the risperidone switch group (*n* = 27). Median percent change in BMD, from open-label baseline to 6-month endpoint (OC) was + 0.4% in the lurasidone continuation group (*n* = 31) and 1.5% in the risperidone switch group (*n* = 13). For the combined treatment groups, 4/44 patients (9.1%) experienced a gain, from open-label baseline to 6-month endpoint, in lumbar spine BMD resulting in a shift in BMD category from osteoporosis to osteopenia, or from osteopenia to normal. A gain in lumbar spine BMD was more common in patients switched from risperidone to lurasidone (15.4% [2/13]) compared with patients continuing lurasidone (6.5% [2/31]). No patient experienced a loss in BMD.

#### Electrocardiographic parameters

There were no clinically meaningful changes in mean ECG parameters during 6 months of open-label treatment with lurasidone. One patient had a QTcF > 500 msec at the month 3 assessment, which represented a ≥ 60-msec increase from open-label baseline; at the next assessment, the patient had a QTcF < 450 msec with a QTcF change score < 60 msec.

#### Physical examination and vital signs

There were no clinically meaningful changes in vital signs (heart rate, systolic and diastolic blood pressure, body temperature) during open-label treatment with lurasidone.

In the subset of patients (*n* = 57) with an ophthalmologic assessment that included dilated funduscopic and slit lamp eye examinations, there were no clinically significant treatment-emergent abnormalities in any ophthalmologic parameter.

### Efficacy

**Fig. 4:**
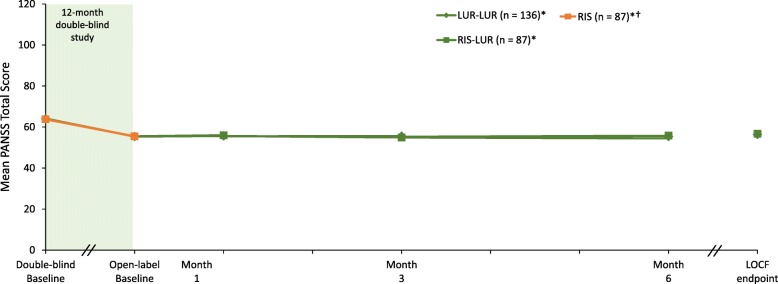
Mean PANSS Total Score From Double-Blind Baseline Through 6 Months of Open-Label Treatment With Lurasidone, by Treatment Assignment in the Double-Blind Study. LUR = lurasidone; PANSS = Positive and Negative Syndrome Scale; RIS = risperidone. ^*^Subgroup entering open-label extension; 6-month completer analysis: LUR-LUR, *n* = 115; RIS-LUR, *n* = 71. ^†^Patients in the RIS-LUR group received risperidone in the 12-month, double-blind study

Patients (per protocol) were clinically stable at entry into the double-blind study (mean baseline PANSS total score of 65.1). At open-label baseline, after completion of 12 months of treatment with lurasidone or risperidone, patients showed improvement in PANSS total score (− 8.7 and − 8.3, respectively). Improvement in PANSS total score was maintained during 6 months of treatment with lurasidone (mean [95%-CI] change from OL baseline to LOCF-endpoint, + 1.0 [− 0.1, + 2.2]). Improvement was maintained on the PANSS total score in both the lurasidone continuation group (+ 1.0 [− 0.5, + 2.6]) and in the risperidone switch group (+ 1.0 [− 0.9, + 2.8]; LOCF-endpoint analysis; Fig. 4). Mean improvement on the CGI-S was also maintained during 6 months of open-label treatment, both in the lurasidone continuation group (0.0 [− 0.1, + 0.2]) and in the risperidone switch group (0.0 [− 0.1, + 0.1]; LOCF-endpoint analysis of change from open-label baseline).

At double-blind baseline, mean MADRS scores were similar for patients randomized to lurasidone and risperidone (6.8 and 6.9, respectively). After completion of 12 months of double-blind treatment with lurasidone or risperidone, mean change scores were − 1.7 and − 2.6, respectively. Mean improvement on the CGI-S was maintained during 6 months of open-label treatment, both in the lurasidone continuation group (+ 0.2 [− 0.6, + 1.0]) and in the risperidone switch group (+ 1.0 [0.1, 2.0]; LOCF-endpoint analysis of change from open-label baseline).

## Discussion

Patients with schizophrenia who completed a previously reported [18] 12-month, double-blind, flexible-dose study of lurasidone versus risperidone, received 6 months of additional open-label lurasidone treatment, with patients in the double-blind risperidone group switching to lurasidone. At the end of the initial 12-month, double-blind phase, treatment with risperidone was associated with statistically significant increases compared to lurasidone in weight, BMI, waist circumference, prolactin levels, glucose, and insulin [18]. After 6 months of treatment in the current extension study, patients who switched from risperidone to lurasidone demonstrated consistent improvement in these safety parameters, with reductions in weight, BMI, waist circumference, glucose, and prolactin levels.

The patient group treated with lurasidone during the initial 12-month study demonstrated consistent improvement from double-blind baseline in weight, BMI, glycemic indices, and metabolic parameters. Small but consistent additional improvement was noted in these parameters during the current 6 months of extension phase treatment with lurasidone.

The weight and metabolic results of the current study are consistent with findings reported from previous lurasidone studies in which long-term treatment with lurasidone was associated with minimal effects on weight, BMI, waist circumference, glycemic indices, and lipid parameters [10–12, 16, 17, 25, 26].

The current results are also consistent with two previously reported lurasidone switch studies. In the first study patients who were treated for 6 weeks with olanzapine showed clinically meaningful reductions in weight, waist circumference, and selected metabolic parameters after switching to 6 months of treatment with lurasidone [17]. In the second study [15] patients (*N* = 240) with a diagnosis of schizophrenia who were stable on treatment with a range of typical and atypical (e.g. olanzapine, quetiapine, risperidone) antipsychotics were switched to lurasidone, 40–120 mg/d. After 6 weeks of open-label treatment with lurasidone, improvement in weight and lipid parameters were observed. In a 6-month, open-label extension of this study, improvements in efficacy on lurasidone were maintained, with minimal long-term effects on weight, metabolic parameters, and prolactin [17].

Among patients in the initial double-blind phase of the current study, treatment with risperidone was associated with notable increases in prolactin levels, with commensurate reduction in prolactin in males (− 11.2 ng/mL) and females (− 30.8 ng/mL) following the switch to lurasidone. Previous systematic reviews and meta-analyses have ranked risperidone and its metabolite paliperidone, in the group most likely to cause hyperprolactinemia, while lurasidone is ranked in the low-risk group [14, 27]. Prolactin-elevating effects of antipsychotics does not appear to be well-correlated with antagonist affinity for the dopamine D_2_ receptor. Lurasidone has slightly higher D_2_ receptor affinity than risperidone (Ki, 1.7 vs. 2.9 [28]. Instead, the brain/plasma concentration ratio, and specifically pituitary D_2_ receptor occupancy, has been reported to be highly correlated with the hyperprolactinemic effects of atypical antipsychotics in patients with schizophrenia [29, 30].

The mean daily dose of lurasidone used during the current 6-month open-label study was 80 mg/d. In the dose range of 40–120 mg/d utilized in the current study, 6 months of treatment with lurasidone was well-tolerated, with a low incidence of parkinsonism (4.5%) and akathisia (3.1%), few adverse events rated as severe (4.9%), and a low rate of discontinuation due to adverse events in both the risperidone to lurasidone switch group (6.9%), and in the lurasidone continuation group (3.7%).

Improvement in psychotic symptoms, as measured by the PANSS total and CGI-S scores, that were observed on both lurasidone and risperidone during the 12-month double-blind phase were maintained after switching to open-label lurasidone.

Notable study limitations include the open-label, non-randomized design, and lack of an active control group. In addition, the sample size in the risperidone switch group that was available at the end of the extension study was relatively small (*n* = 66). While these are common limitations of extension studies, in this instance the limitations are partially mitigated by the initial 12-month lead-in study, which provided a randomized, double-blind comparison of lurasidone and risperidone. We would further note that the completion rate was relatively high (79%) and was similar for both the lurasidone continuation and risperidone switch groups. Finally, it should be noted that enrolment in the initial double-blind study was limited to patients whose psychotic symptoms were clinically stable, and therefore the efficacy results are not generalizable to patients experiencing an acute exacerbation of schizophrenia.

## Conclusion

Relatively little controlled data are available on whether adverse safety effects associated with selected antipsychotics can be reversed by switching medication. The current switch study extends the findings of the previous studies, most of which concerned the weight and metabolic benefit of switching away from olanzapine. The results of this 6-month study suggest that Long-term treatment with lurasidone had minimal effects on body weight, waist circumference, metabolic parameters, and prolactin levels. Patients who switched from risperidone to lurasidone experienced reductions in weight, waist circumference, metabolic parameters and prolactin levels commensurate with increases in these safety parameters experienced during the previous 12 months of treatment with risperidone.

## Data Availability

The dataset used and/or analysed during the current study is available from the corresponding author on reasonable request.

## Abbreviations

AIMS: Abnormal involuntary movement scale
ATP: Adult treatment panel
BARS: Barnes akathisia rating scale
BMI: Body mass index
CGI-S: Clinical global impression, severity
DXA: Dual-energy x-ray absorptiometry
ECG: Electrocardiogram
HbA1c: Glycosylated hemoglobin
LOCF: Last observation carried forward
MADRS: Montgomery-Åsberg depression rating scale
NCEP: National cholesterol education program
OC: Observed case
OL: Open-label
PANSS: Positive and negative syndrome scale
